# Patient and public involvement in care home research: Reflections on the *how* and *why* of involving patient and public involvement partners in qualitative data analysis and interpretation

**DOI:** 10.1111/hex.13269

**Published:** 2021-05-11

**Authors:** Rachel Stocker, Katie Brittain, Karen Spilsbury, Barbara Hanratty

**Affiliations:** ^1^ Population Health Sciences Institute Newcastle University Newcastle upon Tyne UK; ^2^ School of Healthcare University of Leeds Leeds UK

**Keywords:** community participation, long‐term care facilities, patient and public involvement, qualitative research, research design

## Abstract

**Background:**

There is limited evidence for the impact of involving patients and the public (PPI) in health research. Descriptions of the PPI process are seldom included in publications, particularly data analysis, yet an understanding of processes and impacts of PPI is essential if its contribution to research is to be evaluated.

**Objective:**

To describe the ‘how’ of PPI in qualitative data analysis and critically reflect on potential impact.

**Methods:**

We focus on the development and critical reflection of our step‐by‐step approach to collaborative qualitative data analysis (through a series of analysis workshops) in a specific care home study, and our long‐term engagement model with patients and the public (termed PPI partners).

**Results:**

An open access PPI group, with multiple events over time, sustained broad interest in care home research. Recordings of interview clips, role‐play of interview excerpts and written theme summaries were used in workshops to facilitate PPI partner engagement with data analysis in a specific study. PPI resulted in changes to data interpretation and was perceived to make the research process accessible. We reflect on the challenge of judging the benefits of PPI and presenting PPI in research publications for critical commentary.

**Conclusions:**

Patient and public involvement partners who are actively engaged with data analysis can positively influence research studies. However, guidance for researchers is needed on approaches to PPI, including appropriate levels and methods for evaluation. Without more systematic approaches, we argue that it is impossible to know whether PPI represents good use of resources and is generating a real impact.

## 1 BACKGROUND

There is an imperative for researchers to involve patients and the public (PPI) in health and social care studies, with the aim of ensuring relevance to practice. PPI in the UK is defined as research being carried out *with* or *by* members of the public, rather than *to*, *about* or *for* them.^1^ In this context, patients and the public comprise individuals from a wide range of backgrounds, including those with and without specific personal experience of a given health condition, and professionals working in health or social care. Patient and public involvement may be relevant to all stages of the research process, from prioritizing the research questions, design and conduct of the study, through to dissemination of findings.^2^ Funding bodies ask research teams to provide explicit PPI plans in their proposals, which are then judged by panel members, which include experienced PPI representatives. The growth of PPI is underpinned and supported by organizations such as INVOLVE (a UK advisory group) and the US research funder Patient‐Centered Outcomes Research Institute (PCORI).

Evidence for the impact of PPI on the quality, appropriateness and relevance of research is limited, despite the obligations on applied health researchers to incorporate it into their work.^3^, ^4^ Assumptions about the positive benefits of PPI are often implicit in any discussions, and the skills and expertise needed to conduct PPI, or practical challenges involved, are often overlooked.^5^ In many cases, the input of PPI is more visible at the beginning of research, either at the agenda‐setting or at the protocol development stage.^6^ The impact of PPI on later stages of the research process may be more difficult to ascertain.

Although PPI is now an integral, funded component of almost every applied health and social care research project in the UK, its purpose, conduct and impact are surrounded by uncertainty. There are more than 60 published frameworks for PPI^7^ and at least four reviews^8^, ^9^, ^10^, ^11^ of these frameworks. With the exception of a recently published paper that provided guidance for facilitating workshops on PPI involvement and evaluation,^7^ few publications offer any practical guidance on how to involve patients and the public in the research process. Once PPI is planned, it is equally unclear how researchers should go about measuring the effects that PPI has on the process or outcomes of the research study, and the participants.^12^, ^13^ PPI is rarely described in detail in research methods sections of journal publications, so information in the public domain about the conduct of PPI is sparse. This seems to be important, because an understanding of the process of PPI is an essential precursor to understanding and measuring impact. Details of the conduct of PPI in research are needed, in order for PPI to be evaluated in the same way as other complex interventions.^3^

A great deal of PPI in health and social care research has been employed in qualitative studies,^3^, ^5^, ^14^ which may reflect the accessible nature of this research method. A majority of published accounts are from the UK, with studies also originating from the USA, Canada, the Netherlands and Australia.^3^ PPI in the analysis and interpretation of qualitative data is sometimes proposed as best practice.^13^, ^15^ The idea behind collaborative data analysis is that the PPI members will prompt the research team to look at, and understand, the data in new ways,^16^ and this will ultimately lead to an improvement in research accessibility and quality.^17^ Four methodological approaches to involving PPI partners in data analysis were identified in a recent review of collaborative data analysis studies^13^; consultation (researchers conduct the analysis and present their findings to PPI partners for feedback); development (PPI partners are involved in the early stages of analysis); application (PPI partners apply categorized themes to data); and finally a combination of development and application. Engaging PPI partners with collaborative data analysis requires sessions that are more formalized and structured than standard PPI meetings.^13^

In this article, we aim to provide a critical account of the ‘how’ of PPI in our collaborative qualitative data analysis. We use the term ‘PPI partners’ to describe our PPI colleagues involved with our research (eg those with a relative residing in a care home, or involved in working with or in care homes in a voluntary or professional capacity). The term ‘partner’ was specifically selected to highlight the collaborative nature of their involvement. We describe (a) our approach to engaging PPI partners in a portfolio of care home research and (b) our model of collaborative data analysis with PPI partners on a specific study of primary care services for care homes. Whilst our work may offer practical ideas for researchers conducting PPI in health and social care research, we also intend this article to prompt discussion and debate in the research community to develop more understanding of how to promote engagement through guidance, and consider how to measure the impact of PPI for research.

## 2 DESIGN AND SETTING

### 2.1 A Care Home Interest Group

A university‐supported Care Home Interest Group (CHIG) was set up to nurture PPI across a portfolio of planned care home research in the North of England. Recruitment was carried out through local publicity, through direct email contact to local care networks and through the Valuing Our Intellectual Capital and Experience (VOICE) PPI platform (https://www.voice‐global.org).

Membership of this CHIG is dynamic and has grown over time. The group remains active. The group started in 2016 with 33 members: 12 declared no relevant professional experience or qualifications; 10 were health‐care professionals; three were care home staff; three were local authority staff; two were working in clinical governance/research support roles; and three represent charitable/not‐for‐profit organizations. Group meetings are open to anyone with a personal or professional interest in the topic of care homes.

In the early stages of the CHIG, we invited members to align themselves with on‐going care home studies that interested them, and to form interest‐specific study subgroups. Members opted to be involved in none, one or several studies. Formation of a wider interest group was intended to promote the area of research in relevant local communities. It was also intended to be a long‐term initiative, which would not disband once a study was complete, unlike a study‐specific PPI group. Involvement could take a range of forms: face to face at study meetings, and comments on projects via telephone, email or post.

A series of events were held over the first 3 years of the CHIG organization's existence that were open to a wide audience. They included study‐specific PPI events, including for the study detailed below, and wider engagement events. CHIG members also contributed to a UK National Institute for Health Research (NIHR) review of care home research.^18^

### 2.2 Our approach to PPI collaborative qualitative data analysis

In this section, we present and critique our experience of PPI in data analysis for one care home‐related qualitative study. The study aimed to explore perceptions and experiences of primary care services for care homes in the UK from the viewpoint of care home residents, relatives, staff, general practitioners and practice staff, and service commissioners.

Members of the CHIG who had joined this study‐specific PPI group were involved in the study design, protocol development, production of study written materials and interview schedules. Data collected in the study were drawn from over 100 interviews in three distinct geographical areas in England. Collaborative analysis of a data set of this size required a careful approach to ensure we gave our PPI members opportunities to appreciate the data but did not burden them with a task that was unreasonably large. We chose to undertake a preliminary thematic analysis within the research team, which we then presented at PPI meetings. We were careful not to seek validation of our findings. Instead, we invited PPI partners to help us to develop our interpretation and understanding of several of the themes. Our work loosely followed the consultation and application approaches to collaborative analysis, described in Jennings' study.^13^ The two workshops convened to facilitate this process are described in more detail below to offer practical ideas for researchers planning their own PPI collaborative analysis.

### 2.3 Workshop 1

Our first PPI collaborative analysis workshop took place when interviews in the first study site had been completed, transcribed and analysed by the research team. All 11 members of the CHIG subgroup for this study were invited, and seven attended. Before the workshop, anonymized transcript excerpts were selected by the research team. These segments of qualitative data varied in length, and represented either a key theme (from several interviewees) or a range of themes (from an individual interviewee). The themes, under development when shared with PPI partners, included the following: routines of care; issues of power; continuity of care; and working relationships. With each excerpt, we provided some information on the role of the interviewee, size and type of care home or general practice where they worked, and a general idea of geographical location. Written versions of the transcript excerpts were emailed to the attendees in advance of the workshops. Audio recordings of the same data were made to be played in the workshop, with actors voicing the interviewee responses to ensure anonymity. The actors followed, as closely as possible, the verbal nuances of the original interviewee, with guidance from the interviewer. We did not share our ideas about the theme(s) attributed to each excerpt, until the end of the session because we wanted the PPI partners to articulate their interpretation of the data without being influenced by the research team. Box 1 provides an example of the content of an interview audio clip.

BOX 1 **Example of an interview excerpt relating to the theme of organizational ‘routines’**

**Table:** 

Patricia (pseudonym) manages a care home (nursing and residential) in an affluent area of [town]. She is a registered nurse with around 20 years of experience.	
Interviewer:	When you contact these GP surgeries, what kind of proportion of your working day does that tend to take up?
Patricia:	It depends. It's usually a morning task. If anybody's needing to be seen by the GP, the staff write it in the diary. First thing the nurse comes in, when she comes on duty.
Interviewer:	The handover?
Patricia:	Yes, handover and then she'll look in the diary. Takes ten minutes to make the calls, and then when the GP comes, it's just the time off the floor to do that. The only issue is GPs arriving at mealtimes, but we can't do anything about that. They've got to come when it's convenient to them, but we have protected mealtimes. So it means if they arrive at mealtimes, it's taking a member of staff away, off the floor.
Interviewer:	Okay, I see. Okay, so you don't have a specific time that you know the GP will come out?
Patricia:	No.
Interviewer:	Is it any time that day?
Patricia:	It's any time. It's usually around lunchtime, when they finish the morning surgeries.
Interviewer:	Yes, okay. Okay, so they can end up coming in lunchtime? Okay. Okay, how do you deal with that?
Patricia:	There's nothing we can do. The nurse or senior just has to leave the floor and go and deal with the GP. We have to take the residents out of the dining room to go and see them, and that's interrupting their lunch. I don't think there's anything we can do around that, because it's to fit in with the GP’s workload.

A brief update on study progress was given at the beginning of the workshop, followed by an explanation of how we proposed the session would run. Each interview excerpt was presented on paper and via an audio recording. At the end of each excerpt, the PPI group were asked to share any initial thoughts and ideas about what they had just heard. The research team facilitated discussion where necessary, to ensure ideas that supported or challenged the research team's analysis were explored. Contemporaneous notes were made, and discussions were audio‐recorded with permission for later use by the research team.

### 2.4 Workshop 2

A second collaborative data analysis workshop was convened 6 months after workshop 1. Overall study data collection was finished, and a thematic analysis was close to completion. Four PPI partners attended, three of whom had taken part in the first workshop. In this workshop, we employed role‐play to promote discussion of ideas and views of the interview excerpts.

Each PPI partner was given a written interview excerpt aligned to a specific theme, and brief information about the interviewee. An example is provided in Box 2. Name badges showing the role of interviewee (resident/relative/care home staff/GP) were worn. Each PPI partner was asked to read out their excerpt as a role‐play, and when finished, the rest of the group were asked to give their interpretation about what was said—using what they thought would be their point of view, from their own assigned role.

BOX 2 **Examples of an interview excerpt employed in PPI role‐play**

**Table:** 

*Role‐play: Iam a resident of a care home in [area]*. “You're busy having your meal, and somebody will come, a nurse will come and say, “The doctor's here to see you.” “Thank you. Oh, dear”. You just have to leave what you're having for lunch, yes. It's not every time, but if people told me, “The doctor will be coming,” I say, “Well, don't bet on it it's not lunchtime.” I'm sorry, I shouldn't say that.” *Role‐play: Iam a GP at a GP surgery in [area]*. “Traditionally GP practices have always asked, when patients request a visit, please send them in the morning so that we can plan the day. Whereas what happens with the care homes, is that yes, they'll have rung in the morning about one patient, then they'll ring at 3 o'clock in the afternoon about another one. One care home I work with is chaotic, they don't seem to be connected in the different parts of the building, so we'll get calls from one floor in the morning, then half an hour later, or an hour later after someone's been to see the patient we'll get a call for the other floor. It's very difficult.” *Role‐play: Iam a nurse at a care home in [area]*. “The doctor's receptionist will say to me, “Are you going to have all these visit requests in by 11 o'clock ‐ or else! ‐ unless it's an emergency”? It's like, right, so if someone's going to be ill, they'd better make sure they're ill before 11 o'clock. So, what happens if they suddenly start vomiting at 11:10? Oh, how ‘inconvenient’ of them. You know?”

In this workshop, we also allocated some time to focus on one particular theme that had been developed and refined by the research team. A written overview of the theme was presented, with exemplar quotations. We then asked the PPI partners to read this, and share their thoughts on the theme and related ideas, given the data that had been presented. We were seeking confirmation that our interpretation made sense to people with knowledge of the sector, and ideas as to why this theme was so common in our data.

At the end of workshop 2, we dedicated some time to carry out a focus group with our study‐specific PPI partners in attendance, to explore their views on our approach to their involvement in and conduct of the collaborative data analysis workshops. This was classed as a research activity, and the research team recruited and gained written informed consent from our PPI partners to take part in this focus group. Ethical approval to carry out the focus group was provided by Newcastle University Faculty of Medical Sciences Research Ethics Committee (REF: 7102/2018). The focus group was loosely structured as a discussion session, focusing on the methods used in the workshops and opinions on the impact the activity could have had on the analysis and interpretations of data from the associated care home study. These focus group discussions were audio‐recorded and transcribed verbatim (with participants’ permission) by a member of the research team. Data were analysed using a thematic approach.

## 3 FINDINGS

### 3.1 Workshops

In both collaborative data analysis workshops, our PPI partners were from a mix of professional and patient or public backgrounds. This led to challenging and, at times, heated discussion of the themes presented. On several occasions, the non‐professional PPI partners made points that others with health and social care experience were able to reflect on, from their respective roles. The role‐play format appeared to be particularly helpful to prompt appreciation of opposing perspectives and to think about what might be important to the person whose role they were playing.

These workshop interactions presented interpretations of the data that were different from those of the research team. Box 3 shows an example of how the PPI partners reflected on data within our ‘routines’ theme. These discussions led to actions that influenced on‐going data collection and analysis. First, the research team reflected on the workshop discussions and the new interpretations of data offered by PPI partners. We reviewed the interview topic guide and added prompts, to explore issues during subsequent data collection activities with study participants. Our themes and theme descriptors were reviewed through the new lens, and our data interrogated for other examples of issues brought up by PPI partners, such as differing levels of respect for health‐care staff skills, and the role of resident choice in their GP care. We also integrated additional ideas and concepts into the analysis framework for our wider data set. We used role‐play to explore data and key messages and found this format encouraged PPI partners to take different perspectives and consider issues that might be important to the person they were role‐playing.

BOX 3 **Examples of PPI partner discussion of data aligned to a theme of organizational 'routines'**

**Table:** 

• The GP routine and care home routines are not always compatible. Each side has limited resources. Presented as ‘unfortunately just the way it is’ by both sides—a defeatist attitude. If every GP/care home had a bespoke service, it would be chaos. But, there could, and should, be more flexibility in the system. • An important drawback of a flexible, semi‐bespoke working arrangement with a specific GP is how it limits care home residents’ choice of GP surgery/GP. This could further disempower the resident. • There appears to be a tension between *individualised* care, and the organisation of *effective* care, between two separate organisations. Important to remember that a care home is the resident's home, and they should expect the same flexibilities and choice as anyone living in their own home. However, care home staff need to be efficient and organise external health‐care services to offer good overall care, which can limit flexibility and choice. To resolve this, each party needs to understand each other's needs, and be flexible enough to take each situation as it comes, rather than a one‐size‐fits‐all solution that cannot be changed to suit.

### 3.2 PPI participant perspectives on our collaborative qualitative data analysis model, and views on impact on the study

Through the focus group conducted at the end of workshop 2, our PPI partners provided feedback on their experience of participating in the workshops. Our decision to choose specific themes to discuss, rather than presenting full transcripts, was appreciated by most attendees across both workshops. However, the participants also considered whether the research team might have tried to steer the discussion in its choice of data to present.

I wondered if we were being pointed towards a certain way, as you've chosen certain quotes for us which will elicit certain kinds of comments from us. I wondered if there would be a bias creeping in there. Perhaps you could have emailed us whole interviews and had us focus on chunks of it in the meeting. I would be happy to have done that. PPI partner (lay member)

I agree that I'd have been happy to have a whole interview but I'm equally happy with the chunks of transcripts and the way you did it. Because I was given the opportunity to respond, with my opinions and thoughts. And I felt that I was able to speak openly. I didn't feel as if I was being led, I just felt that I was being given the opportunity to speak from my professional and personal experience. And doing it this way [using transcript excerpts, and role‐play] can identify bias and pre‐conceived ideas about certain aspects of health and social care. We do have stereotypical images, and thoughts and approaches. And I certainly feel that my contribution has been valued and taken into account for the analysis. PPI partner (non‐statutory body)

Role‐play was viewed as a useful way of stepping into the mindset of the interviewee and understanding. None of the attendees expressed or showed signs of reluctance to take part.

I enjoyed the way that this [session] has been presented, because I think that having all of us in roles [for the role play session] is useful. Because being in a role places yourself in that mindset, and it's all about the feeling. And it IS about the feeling, it's not about the operation. I think I'd have been really flooded with information if I'd had the full transcript. So having quotes that looked at a particular aspect, was really useful to allow me to focus on that. And for me, sat here, I was thinking, all of the time I'm referring to things I'm thinking of the family member and how they'd feel about this. So it identified some themes for me. PPI partner (charity sector)

## 4 DISCUSSION

We have described an approach to PPI that required investment in time, over a number of years, to develop a group of engaged PPI partners. The techniques that we used to involve these partners with qualitative data analysis are not novel, but they appear to enable PPI to influence our work, without excessive demands on their time outside of scheduled partner meetings. This PPI fulfilled a need for involvement of care recipients, family or members of the public in research, but we make no attempts to judge whether the changes it brought about were appropriate, influential or value for money.

We have presented our approach to PPI in the format of a research paper. This has allowed us to be transparent about not only our processes but also the deficiencies and limitations, and this may leave us open to critical commentary. However, in some studies, one or two individuals contribute to PPI, and commentary on diversity and inclusivity is rare in project reports. Recruitment of our PPI partners was not selective, which caused some discomfort to us as researchers. Similarly, we found our inability to measure the impact of our PPI, or to judge whether it met the level of our peers, to be challenging. As PPI extends from prioritizing questions, into direct input from PPI partners in the conduct of research, the boundaries between PPI activities and research activities appear to be increasingly blurred. This leads us to conclude that practical guidance on its conduct is needed, to add to the existing plethora of publications on principles to underpin PPI.^19^, ^20^, ^21^, ^22^

### 4.1 Comparison with other work

Existing frameworks to support and evaluate patient and public involvement in research are diverse, but mainly used by the groups that develop them.^7^ A lack of transferability leads each group to start afresh and design their own PPI. The authors conclude that a set of resources that can be adapted for local co‐design may be more useful than a single framework. Work to examine best practice in collaborative qualitative analysis has focused on mental health and dementia research.^13^, ^23^ However, PPI was from patients with similar lived experiences. Approaches that are appropriate for a patient group may need modification for work with less involved members of the public. Other work has highlighted some common principles—PPI that is sporadic, rather than throughout the course of a study, may have less impact.^3^

Membership of PPI groups needs thought, particularly the balance between patients who have been service recipients, and members of the public who may have little or no direct experience.^24^ Ethical implications, and the resource and emotional costs of collaborative data analysis with PPI partners also clearly need important consideration.^25^, ^26^

Patient and public involvement within a service setting should have lessons for PPI in research. Much of the work and thinking on PPI in England was stimulated by legislation that placed a statutory duty on National Health Service (NHS) organizations to engage with patients and the public in the evaluation, development and delivery of services (Health and Social Care Act 2001^27^). PPI involvement in this setting has been framed on a continuum, from consultation to partnership and shared leadership, with PPI having differing levels of influence on decision making at each stage.^28^ However, current models of PPI in service improvement are criticized as consultative rather than truly collaborative, and controlled and dominated by professionals.^28^ For example, the formation of patient groups to advise individual general practices, or involvement of public members in the election of hospital governing boards, for example, is beset by all the same questions of representativeness and legitimacy as PPI in research. PPI in research is often more generously funded and, as a result, should be in a position to lead on development of methods and evaluative approaches.

### 4.2 Strengths and weaknesses

At the outset of our work, we sought accounts of what other researchers had done to bring PPI into qualitative research and found a dearth of descriptive work. This paper represents an attempt to redress this balance, with a description of our PPI, both the benefits and challenges. The strengths of our approach are the limited resources and pre‐planning that was needed to conduct our workshops. We did not ask PPI partners to undertake training, use digital tools or software or read lengthy transcripts. Whilst we could be criticized for not contributing to the development of our PPI members, the light‐touch approach was appreciated, and we believe that it helped to maintain input into our work. Further work could be undertaken to maximize the use of online tools for collaborative qualitative data analysis with PPI partners, especially given recent moves towards home‐based working. We do, however, recognize that our approach to PPI is limited, and involvement could be taken further—underpinned by future explicit guidance on PPI practice, which we encourage other researchers to develop and publish. This includes PPI in manuscript writing, which we did not carry out.

We noted an absence (at the time of the study) of any published best practice guides, though a number have since been published, along with an authoritative review of PPI frameworks.^7^, ^13^, ^29^, ^30^ Involvement in this work from care home residents was limited. Engaging care home residents in the research process is often not attempted because it is perceived to be too difficult.^31^ The health and functional status of residents may limit residents’ ability to take part, and if staff are needed to support residents’ participation, their availability may be limited. However, we acknowledge that many of these challenges can be overcome with sensitivity to the demands placed on participants, and careful choice of venue, and methods for presenting materials and demands placed on participants.

### 4.3 Implications

More explicit guidance for researchers is needed on practice and approaches to PPI, including appropriate levels and methods for evaluation. The development of approaches and their evaluation should also involve PPI partners directly. In this study, we allowed our PPI work to impact on our findings, but we have no formal way of judging whether this was appropriate. However, we consider that our collaborative qualitative data analysis with our PPI partners added value, and promoted the resonance and relevance of study findings. Without a more systematic approach, and willingness to publish details of the process undertaken, it will be impossible to design appropriate evaluations. Now that PPI is an accepted responsibility for a research team, the research community should commit to developing evaluative approaches to PPI, to ensure it does represent good use of resources and generates meaningful impact.

## CONFLICT OF INTEREST

The author(s) declare that there is no conflict of interest.

## PATIENT OR PUBLIC CONTRIBUTION

Members of the public and caregivers were involved in the design, conduct and interpretation of the data in this study.

## Data Availability

The data that support the findings of this study are available on request from the corresponding author. The data are not publicly available due to privacy or ethical restrictions.
